# Identification and characterization of miRNAs associated with sterile flower buds in the tea plant based on small RNA sequencing

**DOI:** 10.1186/s41065-021-00188-8

**Published:** 2021-07-16

**Authors:** Hao Qu, Yue Liu, Huibing Jiang, Yufei Liu, Weixi Song, Linbo Chen

**Affiliations:** 1 Tea Research Institute, Yunnan Academy of Agricultural Sciences, Menghai, Xishuangbanna, 666201 China; 2 Yunnan Provincial Key Laboratory of Tea Science, Menghai, Xishuangbanna, 666201 China

**Keywords:** miRNA, *Camellia sinensis*, Sterile floral bud, Target genes

## Abstract

**Background:**

miRNAs are a type of conserved, small RNA molecule that regulate gene expression and play an important role in the growth and development of plants. miRNAs are involved in seed germination, root development, shoot apical meristem maintenance, leaf development, and flower development by regulating various target genes. However, the role of miRNAs in the mechanism of tea plant flower sterility remains unclear. Therefore, we performed miRNA sequencing on the flowers of fertile male parents, female parents, and sterile offspring.

**Results:**

A total of 55 known miRNAs and 90 unknown miRNAs were identified. In the infertile progeny, 37 miRNAs were differentially expressed; 18 were up-regulated and 19 were down-regulated. miR156, miR157, miR164, miR167, miR169, miR2111 and miR396 family members were down-regulated, and miR160, miR172 and miR319 family members were up-regulated. Moreover, we predicted that the 37 differentially expressed miRNAs target a total of 363 genes, which were enriched in 31 biological functions. We predicted that miR156 targets 142 genes, including *ATD1A*, *SPL*, *ACA1*, *ACA2*, *CKB22* and *MADS2*.

**Conclusion:**

We detected a large number of differentially expressed miRNAs in the sterile tea plant flowers, and their target genes were involved in complex biological processes. Among these miRNAs, the down-regulation of miR156 may be one of the factor in the formation of sterile floral buds in tea plants.

**Supplementary Information:**

The online version contains supplementary material available at 10.1186/s41065-021-00188-8.

## Introduction

Tea is one of the most popular and widely consumed beverages in the world. As such, producers expect tea varieties to have high yield [1]. However, the process of tea plant flowering indicates that the energy of nutrition turns to reproductive growth, which will compete for nutrition and lead to a reduction in production. Therefore, investigating the mechanism of tea plant sterility may play a key role in increasing the yield of tea. Among the factors that cause flower sterility, the abortion or degradation of stamens and pistils is an important aspect that causes plants to produce sterile flowers, and its molecular mechanisms have attracted much attention [2].

MicroRNAs (miRNAs) are a type of endogenous, non-coding, single-stranded RNA molecule composed of about 21 to 25 nucleotides (nt) in length that exist widely across organisms and are quite conserved [3]. Generally, miRNAs recognize their target gene mRNAs through complementary pairing with the mRNA, and degrade or inhibit the translation of the mRNA, thereby regulating the abundance and function of the target mRNA [4]. A miRNA can usually regulate multiple target genes, and the same target gene may also be regulated by multiple miRNAs [5]. Researchers have discovered that miRNAs are involved in regulating plant biological processes, such as leaf development [6], anther development [7], cell differentiation [8], flowering [9], floral organ morphology development [10], and response to environmental stress [11]. In addition, mutations in miRNA target genes cause defects in plant development [12], indicating that miRNAs play an important role in promoting plant survival.

Studies have shown that a number of miRNAs, including miR156, miR159, miR160, miR164, Mir166/165, miR167, miR169, miR172, miR319, miR390, and miR399, are related to flower development. It has been reported that miR156, miR172 and miR390 are involved in the regulation of flowering time; miR156 was up-regulated at the juvenile stage to prevent flowering, followed by a decrease in expression with plant growth [13]; miR172 acts downstream of miR156, *SPL9* and *SPL10*, and was regulated by miR156, contrary to the expression pattern of miR156 [14]; mutations in miR164 affected the development of the carpel [15]; miR167 can regulate the expression of *ARF6* and *ARF8* in specific floral organs, but different isoforms showed differences in the ability to inhibit *ARF6* and *ARF8* expression [16]. Similar to miR159, up-regulation of miR319 causes stamen defects, leading to sterility [17]. Moreover, auxin response elements have been shown to up-regulate the expression of miR160, while miR160 down-regulated the expression of the genes *ARF10*, *ARF16* and *ARF17* [18].

In our study, we selected Foxiang2 (FBH), Fudingbaicha (MBH), and their hybrid sterile progeny (ZDH) because of the self-incompatibility of tea plants. We performed small RNA sequencing on flowers, analyzed the differentially expressed genes, and predicted the target genes of different miRNAs. Through the analysis of the data, we identified the key miRNAs related to tea plant sterility and provided reference information for research on the mechanism of tea plant sterility .

## Materials and methods

### Plant materials

**Fig. 1:**
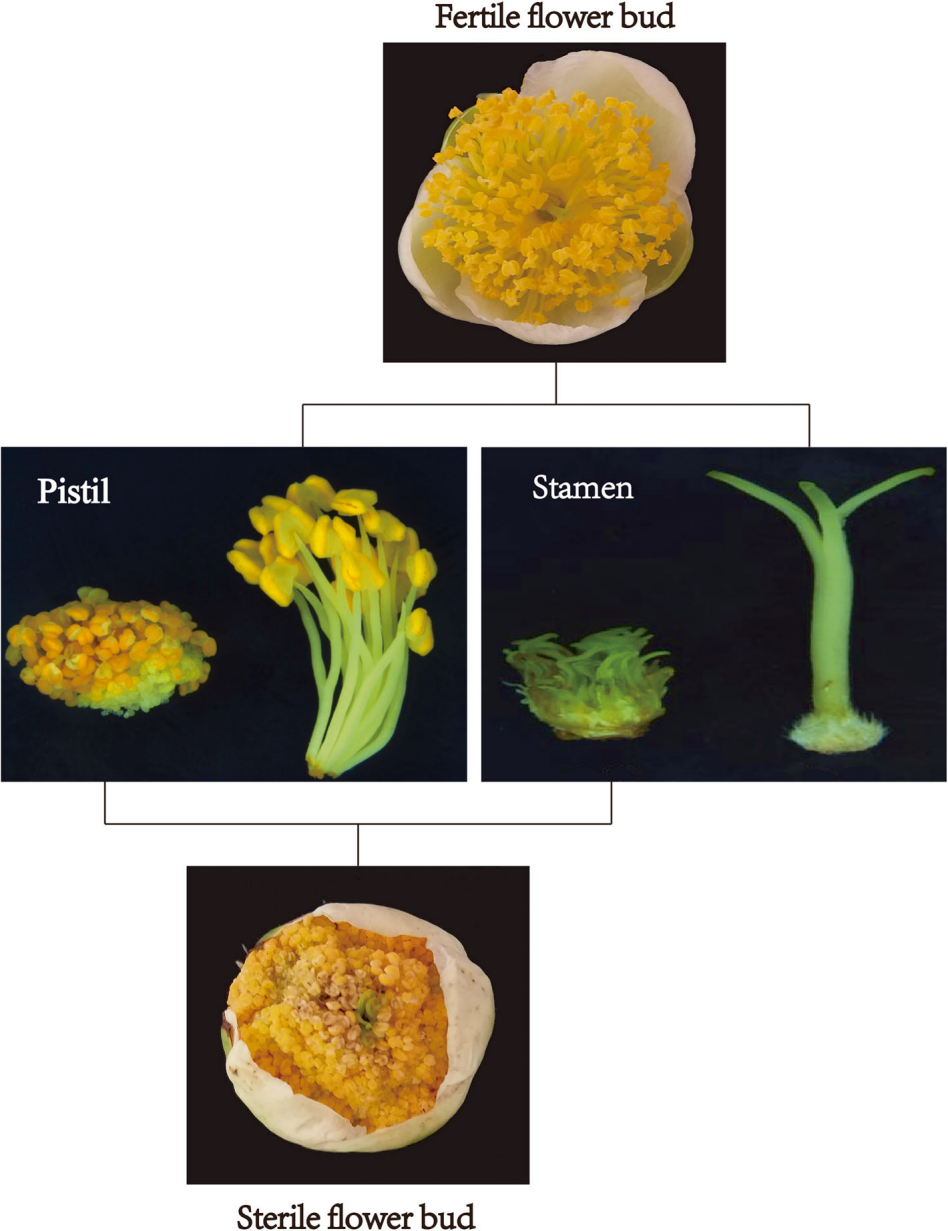
Morphological characteristics of the tea flower

The tea samples (*C. sinensis (L.) O. Kuntze*) used in this experiment were ‘Foxiang2’ (FBH), ‘Fudingbaicha’ (MBH) and sterile hybrid (ZDH), were collected from the Tea Research Institute of Yunnan Academy of Agricultural Sciences. The floral organ of ‘Foxiang2’ and ‘Fudingbaicha’ were complete bisexual flowers. The hybrid offspring of sterile flower buds consist of multiple dwarf pistils, the petals cannot expand normally, the filaments were short, and no pollen in the anthers (Fig. 1). The buds of the samples were collected during the flowering period, and three biological replicates were obtained in each group. The samples were frozen in liquid nitrogen and refrigerated at − 80 °C.

### RNA library construction and sequencing

Small RNA library construction and sequencing were conducted by Novogene Technology (Beijing, China). The Small RNA Sample Pre-Kit was used to construct the library. Briefly, with total RNA as the starting material, the special structure of the 3′ and 5′ ends of Small RNA were used to add linkers to both ends of the Small RNA, followed by reverse transcription to synthesize cDNA to construct the library. The effective library concentration was greater than 2 nM. Agilent 2100 was used to detect the insert size of the library and Illumina HiSeq 2000 sequencing was performed. All of the experiments were performed in triplicate. In order to ensure the quality of information analysis, the following was removed: low-quality reads, reads with less than 10% base information, reads contaminated with 5′ linkers, reads without 3′ linker sequences and inserts, 3′ linker sequences, and polyA/T/G/C reads.

### miRNA analysis

The Bowtie software package locates the small RNA after length screening to the reference sequence, and analyzes the distribution of small RNAs on the reference sequence [19]. Then, the reads mapped to the reference sequence are compared with the specified range sequence in miRBase to obtain the details of the matched small RNA of each sample, including the secondary structure of the known miRNA on the match, the sequence of the miRNA in each sample, and information, such as length and number of occurrences. The signature hairpin structure of miRNA precursors was used to predict new miRNAs. The comparison and annotation of all small RNAs and various RNAs were then summarized. Total rRNA in the classification annotation results can be used as a quality control standard for a sample, and the proportion of the total rRNA in plant samples should be less than 60%.

### Prediction and analysis of target genes

To obtain the corresponding relationship between miRNA and target gene, the target genes of known and novel miRNAs were predicted using the miRNA target gene prediction software psRobot [20]. Gene Ontology (GO) enrichment analysis was performed on each set of differentially expressed miRNA target genes. GO enrichment analysis was based on Wallenius non-centralhyper-geometric distribution.

### Analysis of differentially expressed miRNAs

We performed statistical analysis on the expression of known and new miRNAs in each sample. The input data of differential expression is the read count data obtained from miRNA expression level analysis. For the biological replicates, DESeq2 based on negative binomial distribution was used for analysis. In the comparison of the two samples, the binomial distribution was emphasized, and A1 and A2 were the total number of reads. Define M = (log2A1-log2A2) and A = (log2A1 + log2A2/2). DESeq2 calculates the difference expression based on the MA-plot [21]. The resulting *P*-values were adjusted using the Benjamin and Hochberg approach for controlling the false discovery rate. The genes with *P*-value < 0.05 found by DESeq were differentially expressed [22].

### Quantitative real-time PCR assays

Total RNA was isolated using TRlpure reagent (BioTeke, China) according to the manufacturer’s instructions. cDNA was synthesized from total RNA using a PrimeScript RT reagent kit (TaKaRa, Japan). The obtained cDNA was used as a template in a SYBR green-basedq-PCR reaction kit (CFX-96, Bio-Rad, Hercules, CA, USA). miR165a-3p was used for normalization, and the primers are shown in Table 1.

**Table 1:** Primer Sequences for q-PCR

miRNA name	Primer Sequence (5′ to 3′)
miR172e-3p	F:ACACTCCAGCTGGGGGAATCTTGATGAT
	R:CTCAACTGGTGTCGTGGAGTCGGCAATTCAGTTGAGATGCAGC
miR172a	F:ACACTCCAGCTGGGAGAATCTTGATGAT
	R:CTCAACTGGTGTCGTGGAGTCGGCAATTCAGTTGAGATGCAGC
miR164a	F:ACACTCCAGCTGGGTGGAGAAGCAGGGC
	R:CTCAACTGGTGTCGTGGAGTCGGCAATTCAGTTGAGTGCACGT
miR164c-5p	F:ACACTCCAGCTGGGTGGAGAAGCAGGGC
	R:CTCAACTGGTGTCGTGGAGTCGGCAATTCAGTTGAGCGCACGT
miR160a-5p	F:ACACTCCAGCTGGGTGCCTGGCTCCCTG
	R:CTCAACTGGTGTCGTGGAGTCGGCAATTCAGTTGAGTGGCATA
miR156a-5p	F:ACACTCCAGCTGGGTGACAGAAGAGAG
	R:CTCAACTGGTGTCGTGGAGTCGGCAATTCAGTTGAGGTGCTCA
miR156j	F:ACACTCCAGCTGGGTGACAGAAGAGAG
	R:CTCAACTGGTGTCGTGGAGTCGGCAATTCAGTTGAGGTGCTCT
miR157d	F:ACACTCCAGCTGGGTGACAGAAGATAG
	R:CTCAACTGGTGTCGTGGAGTCGGCAATTCAGTTGAGGTGCTCT
miR157a-5p	F:ACACTCCAGCTGGGTTGACAGAAGATAG
	R:CTCAACTGGTGTCGTGGAGTCGGCAATTCAGTTGAGGTGCTCT
miR319c	F:ACACTCCAGCTGGGTTGGACTGAAGGGA
	R:CTCAACTGGTGTCGTGGAGTCGGCAATTCAGTTGAGAAGGAGC
miR319a	F:ACACTCCAGCTGGGTTGGACTGAAGGGA
	R:CTCAACTGGTGTCGTGGAGTCGGCAATTCAGTTGAGAGGGAGC
novel_126	F:ACACTCCAGCTGGGCATCGAAATCACCAG
	R:CTCAACTGGTGTCGTGGAGTCGGCAATTCAGTTGAGCTCACAT
pc-3p-222	F:ACACTCCAGCTGGGTTTCCAAGACCACCC
	R:CTCAACTGGTGTCGTGGAGTCGGCAATTCAGTTGAGTCGGCAT
All	R:TGGTGTCGTGGAGTCG

## Results

### Small RNA sequencing data

**Fig. 2:**
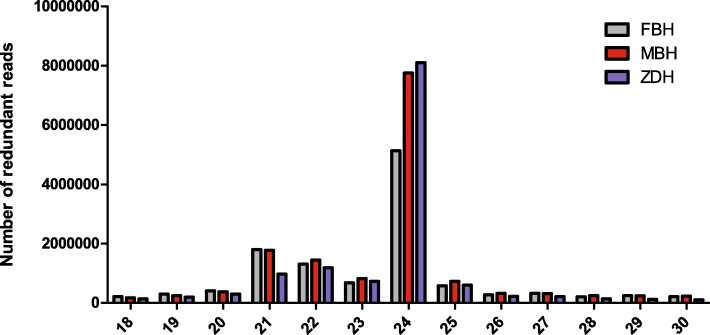
Distribution of small RNA length. The abscissa represents the number of bases, and the ordinate represents the number of redundant reads

In order to explore the role of miRNAs in the regulation of tea plant flower development, we used high-throughput sequencing to construct miRNA libraries of FBH, MBH and ZDH. The Q20 percentage of raw data exceeded 98%, and the Q30 percentage was above 96%, with the ratios of GC content above 47%. As a result, a total of 13,976,247, 16,644,199, and 13,976,692 clean reads were obtained for FBH, MBH and ZDH, respectively. The length of plant small RNA ranged from 18 ~ 30 nt, and a total of 11,704,253, 14,693,758 and 13,045,116 small RNA sequence read were identified for FBH, MBH and ZDH, respectively (Table 2). The results showed that the small RNA in the three miRNA libraries were mainly distributed in the range of 21 ~ 24 nt, of which 24 nt was the most abundant (Fig. 2). In regard to the position of the small RNA after length screening to the reference sequence, the ratio of mapped small RNA for for FBH, MBH and ZDH was 60.49, 53.61 and 56.4%, respectively. The number of known miRNAs were 108,703, 134,730 and 105,547, novel miRNA were 64,681, 74,929 and 53,515, rRNA were 411,500, 420,492 and 295,026, ta-siRNA were 33,333, 40,746 and 21,517, snRNA were 8441, 8086 and 2975, and snoRNA were 4114, 3020 and 3227, resoectively (Table 3).

**Table 2:** Summary Dataset of small RNA and transcriptome libraries

Category	male parent (FBH)	female parent (MBH)	sterile flowers (ZDH)
Raw reads	14,270,452	16,985,205	14,315,444
Clean reads	13,976,247	16,644,199	13,976,692
GC content	48.74%	47.94%	47.24%
Q20	98.35%	98.17%	96.73%
Q30	97.14%	96.84%	96.73%
Total sRNA	11,704,253	13,045,116	14,693,758

**Table 3:** small RNA classification statistics

types	male parent (FBH)	female parent (MBH)	sterile flowers (ZDH)
Mapped sRNA	7,079,588 (100.00%)	8,286,830 (100.00%)	6,993,877 (100.00%)
Known_miRNA	108,703 (1.54%)	134,730 (1.63%)	105,547 (1.51%)
rRNA	411,500 (5.81%)	420,492 (5.07%)	295,026 (4.22%)
tRNA	2 (0.00%)	4 (0.00%)	0 (0.00%)
snRNA	8441 (0.12%)	8086 (0.10%)	2975 (0.04%)
snoRNA	4114 (0.06%)	3020 (0.04%)	3227 (0.05%)
novel miRNA	64,681 (0.91%)	74,929 (0.90%)	53,515 (0.77%)
ta-siRNA	33,333 (0.47%)	40,746 (0.49%)	21,517 (0.31%)
Others	6,448,814 (91.09%)	7,604,823 (91.77%)	6,512,070 (93.11%)

### Screening and analysis of the miRNAs

**Fig. 3:**
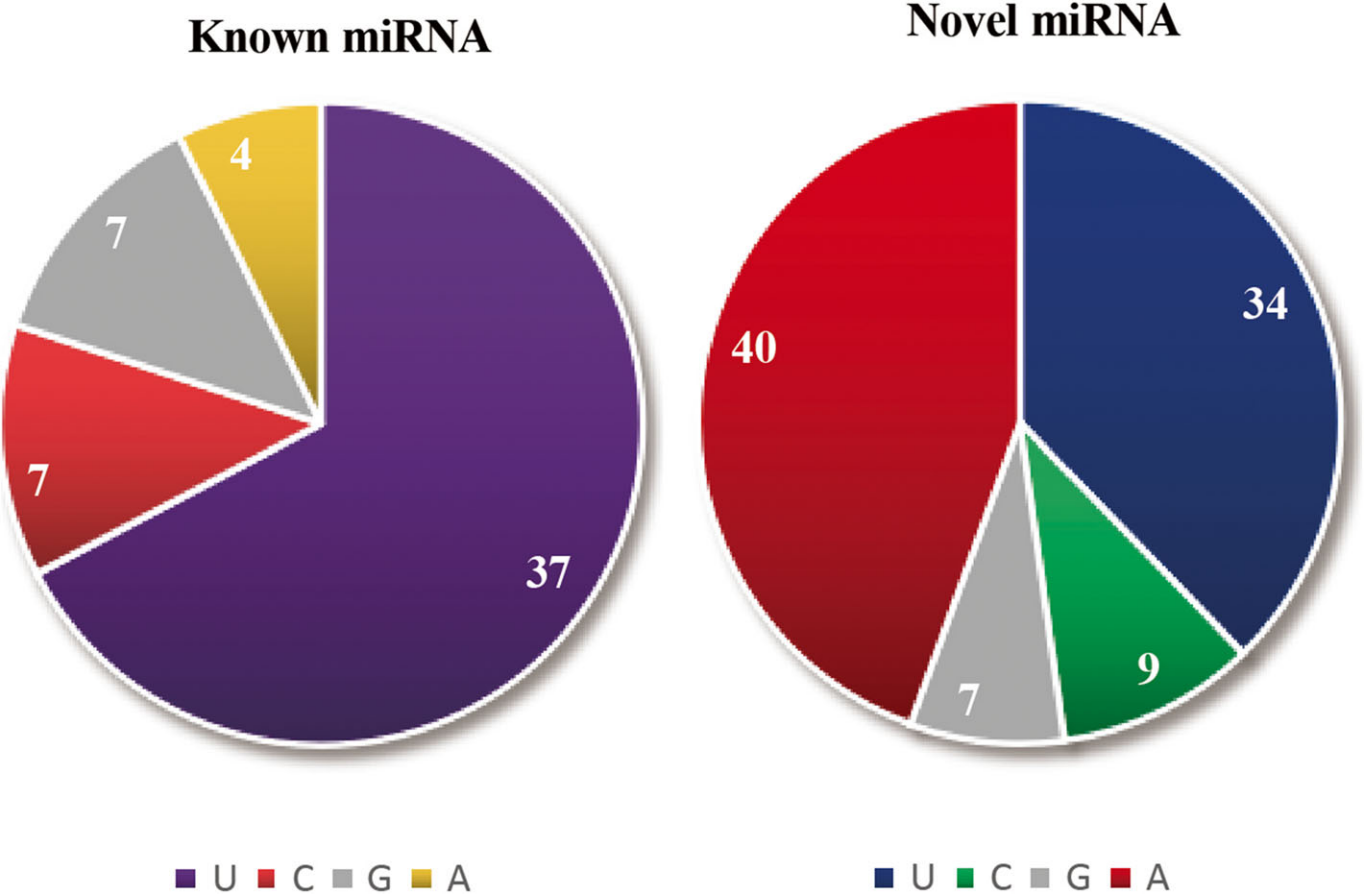
The number of bases in the first position of the miRNA sequence. Among the known miRNAs, there were 37 miRNAs whose first base was U, 7 with C, 7 with G, and 4 with A. Among the novel miRNAs, there were 40 miRNAs whose first base was A, 34 with U, 9 with C, and 7 with G

To identify known miRNAs, we compared our data with known miRNA data of *Arabidopsis* in the miRBase 21.0 database (http://www.mirbase.org/ftp.shtml). A total of 55 known miRNAs were identified and divided into 27 miRNA families. Among these miRNA families, miR156 and miR396 contained four members, miR159, miR166, miR169, miR172 and miR399 contained three members, miR157, miR160, miR164, miR167, miR169, miR170, miR171, miR319, miR390, miR395, miR398 and miR858 contained 2 members, and miR162, miR165, miRK2111, miR393, miR394, miR403, miR408 and miR8175 contained 1 member (Table S1). In addition, we identified 90 putative novel miRNAs (Table S2). All novel miRNA sequences were 19 to 25 nt in length, of which the largest proportion, of miRNAs, accounting for 40.00%, were 24 nt. Interestingly, the sequence with A as the first base accounted for 44% of the novel miRNAs, and the sequence with U as the first base comprised the largest proportion of known miRNAs (Fig. 3).

### Differentially expressed miRNAs

**Fig. 4:**
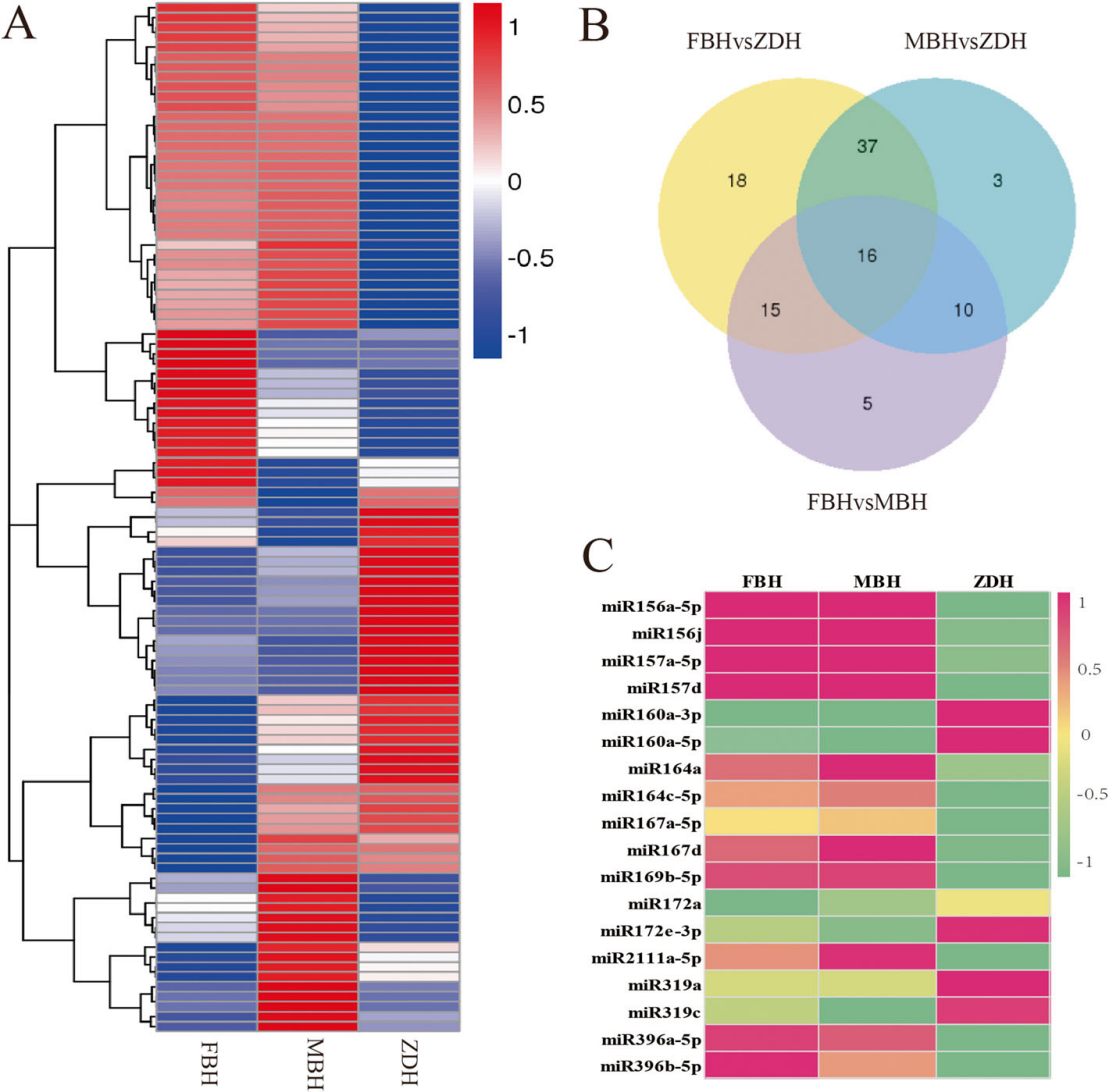
Analysis of differentially expressed miRNAs. **A** miRNA cluster analysis, clustering by log10(TPM + 1) value, where red indicates high expression miRNA and blue indicates low expression miRNA. **B** Venn diagram showing the number of differentially expressed miRNA. **C** Relative expression heat map of known miRNAs, where pink represents high expression and green represents low expression

The expression level of the known and new miRNA in each sample was measured, and the expression level was normalized with TPM [23]. A total of 104 differentially expressed miRNAs were detected in FBH, MBH and ZDH (Fig. 4A). Venn diagram analysis revealed the unique and shared miRNAs in the samples (Fig. 4B). There were 86 differentially expressed miRNAs in common between FBH and ZDH, and 66 differentially expressed miRNAs in common between MBH and ZDH. Notably, 37 differentially expressed miRNAs identified in ZDH, and their expression was significantly different from FBH and MBH, but there was no difference between FBH and MBH. Among these 37 miRNAs, 18 miRNAs were up-regulated and 19 miRNAs were down-regulated. Twelve of the miRNAs that were down-regulated included miR156, miR157, miR164, miR167, miR169, miR2111 and miR396 family members, and 6 of the up-regulated miRNAs included miR160, miR172 and miR319 family members (Fig. 4C) (Table 4).

**Table 4:** Differentially expressed miRNA

miRNA	RPM (FBH)	RPM (MBH)	RPM (ZDH)	ZDH vs FBH	ZDH vs MBH
ath-miR156a-5p	605.19	492.24	16.74	down	down
ath-miR156j	314.01	305.67	27.90	down	down
ath-miR157a-5p	1941.16	1845.92	72.55	down	down
ath-miR157d	45.67	35.73	0.00	down	down
ath-miR160a-3p	0.00	0.00	11.16	up	up
ath-miR160a-5p	274.05	194.52	1679.73	up	up
ath-miR164a	856.40	1004.34	396.22	down	down
ath-miR164c-5p	770.76	829.67	284.61	down	down
ath-miR167a-5p	6257.40	7034.34	1618.35	down	down
ath-miR167d	23,596.55	31,491.75	1819.24	down	down
ath-miR169b-5p	11.42	11.91	0.00	down	down
ath-miR172a	17.13	47.64	94.87	up	up
ath-miR172e-3p	62.80	27.79	217.64	up	up
ath-miR2111a-5p	5.71	7.94	0.00	down	down
ath-miR319a	99,045.01	99,008.67	250,503.15	up	up
ath-miR319c	6645.63	4354.78	15,190.13	up	up
ath-miR396a-5p	14,181.91	12,988.91	3833.80	down	down
ath-miR396b-5p	149,720.80	107,615.02	44,387.31	down	down

### Analysis of miRNA target genes

In order to investigate the regulatory effect of the miRNAs on gene expression, the psRobot software was used to predict the target genes by analyzing the known and novel miRNAs. A total of 145 miRNAs were analyzed and 4007 genes were predicted. Among the predicted genes, 1200 genes were annotated, including *WDR*, *MYB*, *bHLH*, *SPL*, *WRKY*, *NAC*, *APETALA2*, *AGL*, *ARF*, *AP2*, as well other transcription factors. The results indicated that miRNAs target multiple genes, and one gene is targeted by multiple miRNAs, suggesting that miRNAs can regulate multiple functions, and that miRNAs have the same or different regulatory effects in tea plants.

**Fig. 5:**
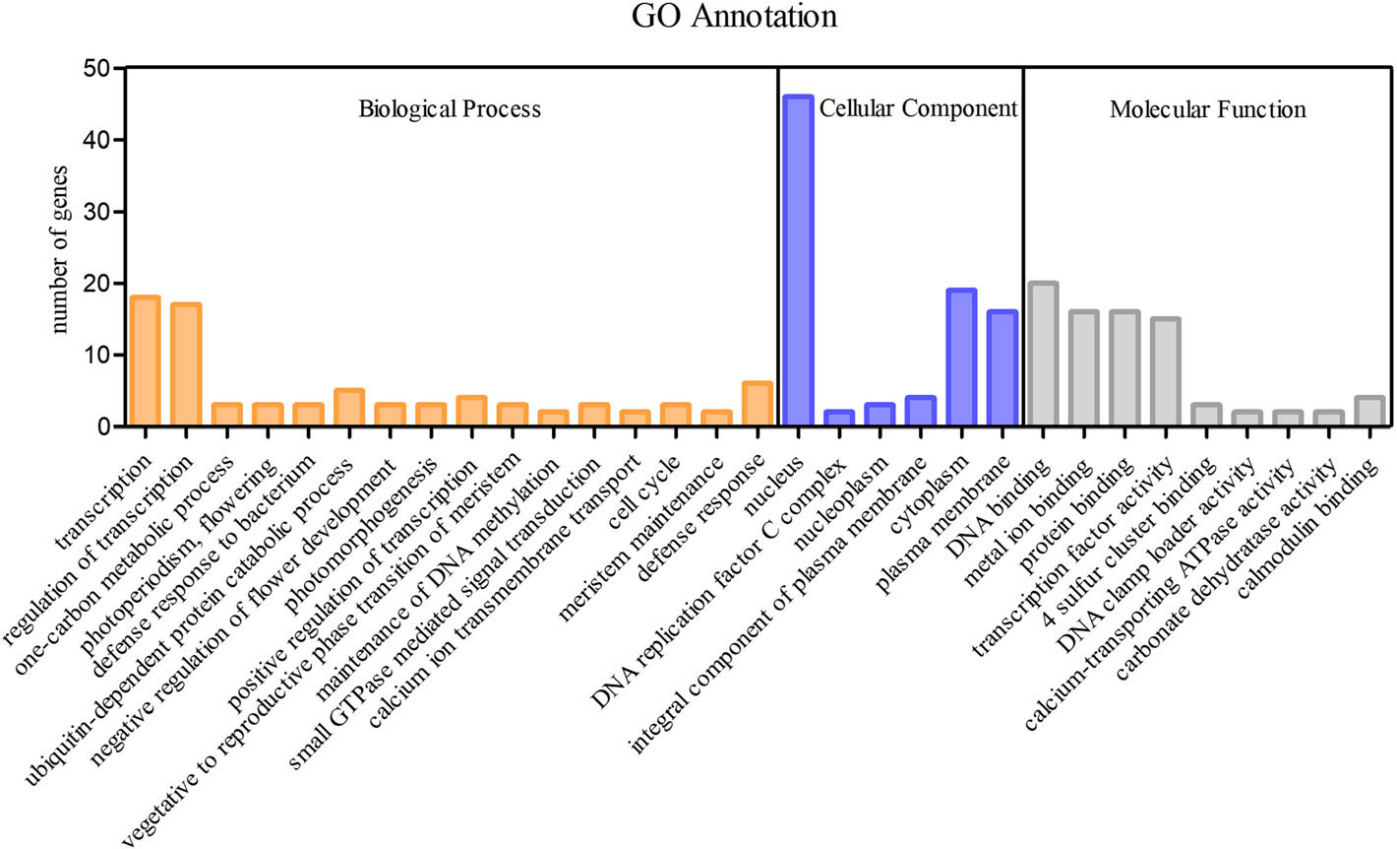
GO enrichment analysis of the differentially expressed miRNA. Orange indicates biological processes, blue indicates cellular components, and gray indicates molecular functions

In addition, 37 differentially expressed miRNAs have 363 annotated target genes. In order to study the function of miRNAs further, the characteristics of the 363 target genes were analyzed through the GO database, including three biological processes, cellular components, and molecular function. The results showed that a total of 31 subcategories were enriched, and “transcription” and “regulation of transcription” were the most enriched biological process. Cellular component analysis showed that “nucleus”, “cytoplasm” and “plasma membrane” were the most representative subcategories. “DNA binding”, “metal ion binding”, protein binding” and “transcription factor activity” were the most representative groups of molecular function. Furthermore, 3, 3, 2, and 4 genes were enriched in “photoperiodism/flowering”, “negative regulation of flower development”, “calcium ion transmembrane transport”, and “calmodulin binding”, respectively (Fig. 5).

Among the target genes, three auxin response factor genes (*ARF18*) targeted by miR160a-5p were predicted; nine floral homeotic protein (*AP2*) and two ethylene-responsive transcription factor (*RAP2–7*) co-targeted by miR172a, miR172c and miR172e-3p were predicted; two transcription factors (*GAMYB*) targeted by miR319c were predicted; two calcium-transporting ATPases (*ACA12*) co-targeted by miR396a-5p and miR396b-5p were predicted; and one ABC transporter I family member (*ABCI11*) co-targeted by miR172a and miR172c were predicted. In addition, we predicted that miR156 has 142 target genes, including three ATPase family AAA domain-containing proteins (*ATD1A*), four Squamosa promoter-binding proteins (*SPL*), two calcium-transporting ATPases (*ACA1* and *ACA2*), two Cyclin-dependent kinases (*CKB22*), and Floral homeotic protein (*MADS2*). These results indicated that the differentially expressed miRNAs regulate complex biological processes.

### qPCR analysis of miRNA

**Fig. 6:**
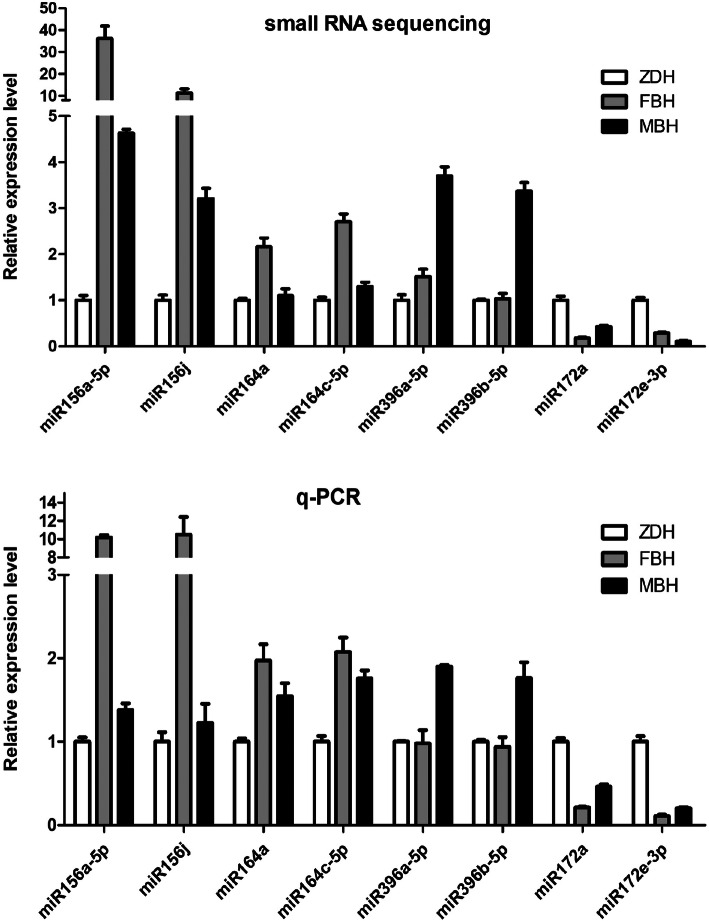
qPCR analysis of selected differentially expressed miRNAs. Data is presented as the mean ± SD, and *n* = 3 independent experiments

We randomly selected 12 miRNAs for RT-qPCR analysis to verify the accuracy of the small RNA sequencing. Among these miRNAs, miR156a-5p, miR156j, miR157a-5p, miR157d, miR164a and miR164c-5p were down-regulated, and miR319, amiR319, cmiR172a, miR172e-3p, miR160a-5p and novel_126 were up-regulated in ZDH (Fig. 6). The expression level of these miRNAs in FBH, MBH and ZDH was consistent with the change trend of small RNA sequencing analysis.

## Discussion

As the reproductive structure of plants, flowers are the key to species reproduction. Flower development is regulated by external factors, such as the environment, and the plant’s genetic information [24]. MicroRNAs play an important role in gene regulation and participate in various biological functions by degrading target gene transcripts or disturbing translation [25]. Here, we selected FBH, MBH and ZDH for small RNA sequencing analysis. By narrowing the scope of differentially expressed miRNAs, the miRNAs related to infertility can be accurately discovered. A total of 55 known miRNAs and 90 novel miRNAs were identified, and 4007 target genes were predicted. Additionally, 37 differentially expressed miRNAs were screened and 363 miRNA target genes were predicted. Among them, miR156, miR160, miR164, miR167, miR169, miR172 and miR319 families have been confirmed to be involved in regulating the formation and development of floral organs.

In *Arabidopsis*, miR160 participates in the auxin-related signal transduction pathway through targeted regulation of the *ARF10*, *ARF16* and *ARF17* genes, and regulates the growth and development of flowers [26]. miR172 controls sex differentiation and meristem cells by targeting AP2-like [27, 28]. Overexpression of miR172 can reduce the level of AP2 protein and also produces abnormal flower phenotypes [29]. The absence of miR319a cause defects in the development of petals and stamens, such as abnormal petals and impaired anther formation [30]. In addition, miR319 regulates the morphology of petals and stamens by regulating the TCP family [31]. Overexpression of the *TCP24* gene in *Arabidopsis* destroys the cell wall of anther endoderm hierarchy, leading to male sterility [32]. In contrast, the expression of miR160, miR172 and miR319 in ZDH was significantly up-regulated. As such, we concluded that the up-regulation of miR160, miR172 and miR319 may cause tea plant sterility.

miR156 inhibits the expression of the SPL transcription factor family to regulate the formation and development of floral organs [33], and 11 genes in the SPL family are regulated by miR156. A previous study reported that miR156 controls the elongation of *Arabidopsis* petals and sepals by regulating the expression of the *SPL2* gene [34]. *SPL8* gene deletion in *Arabidopsis* results in small anthers, less pollen, and reduced fertility [35]. Notably, miR172 influences floral organ identity by regulating *AP2* [36], and miR156 positively regulates the expression of miR172 [37]. Our results revealed that miR156 and miR172 had opposite expression patterns in ZDH compared with FBH and MBH, suggesting that miR156 and miR172 are involved in another regulatory mechanism in producing sterile tea plant flowers. In addition, miR164 targets NAC transcription factors, including *CUC1* and *CUC2*, which can promote the development of floral organs [38]. In double mutants of *CUC2* and *CUC1*, floral organ fusion occurred, and the number of pistil and stamen were reduced [39]. We identified that the expression of miR156 and miR164 families were down-regulated in sterile flowers, indicating that miR156 and miR164 also play a key role in the mechanism of sterility.

miR159 and miR319 have overlapping effects in the regulation of flower development, regulating flowering time and anther development [17]. In addition, miR2118 also regulates the morphology and development of anthers [40]. In our results, the miR159 and miR2118 families, which have been reported to be related to flower development, were not differentially expressed. This indicates that they were not involved in the formation of sterile floral buds in this study.

**Fig. 7:**
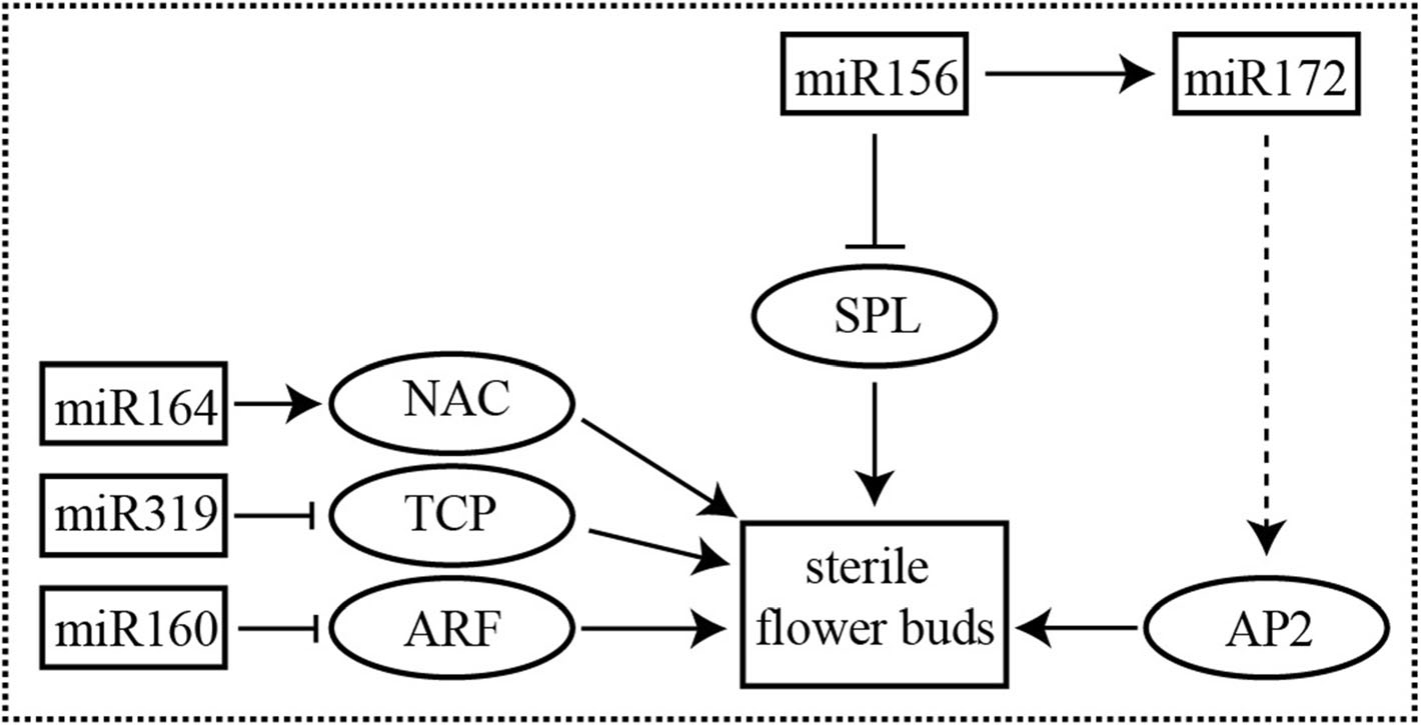
Schematic representation of the pathway for sterile floral buds in tea plants

In conclusion, Fig. 7 showed a schematic representation of the proposed molecular basis of the above results and published literature [41] on sterile floral buds. According to previous studies, we found that the SPL family of genes were up-regulated in ZDH [41]. Combined with the results, We hypothesize that the down-regulation of miR156 and the decreased inhibitory effect lead to overexpression of *SPL.* In contrast to the previous reports, up-regulation of miR172 did not inhibit *AP2* expression. In addition, miR160, miR164, and miR319 were also involved in regulating the formation of sterile floral buds. Our study identified a number of important miRNAs related to tea plant fertility, these results provided a basis for further elucidating the mechanism of sterile floral buds in tea plant.

## Supplementary Information

**Additional file 1: Table S1.** Identification of known miRNAs. **Table S2** Identification of novel miRNAs.

## Data Availability

We have provided detailed information about the materials and methods in our manuscript.

## Abbreviations

FBH: Foxiang2
MBH: Fudingbaicha
ZDH: Hybrid sterile flowers
GO: Gene ontology
KEGG: Kyoto encyclopedia of genes and genomes
Q20: Quality score 20
TPM: Transcripts per million clean tags
