# Supplementary material for: Identification and characterization of miRNAs associated with sterile flower buds in the tea plant based on small RNA sequencing
Source: Hereditas. 2021 Jul 16;158:26. doi: 10.1186/s41065-021-00188-8 (PMC8285856; doi:10.1186/s41065-021-00188-8)
Supplement: Supplementary file 1 — Additional file 1: Table S1. Identification of known miRNAs. Table S2 Identification of novel miRNAs. [file 41065_2021_188_MOESM1_ESM.doc]

**Table S1 Identification of known miRNAs**

| miRNA family | miRNA name | Sequence (5′-3′) | Length (nt) |
| --- | --- | --- | --- |
| miR156 | ath-miR156a-5p | UGACAGAAGAGAGUGAGCAC | 20 |
|  | ath-miR156g | CGACAGAAGAGAGUGAGCAC | 20 |
|  | ath-miR156i | UGACAGAAGAGAGAGAGCAG | 20 |
|  | ath-miR156j | UGACAGAAGAGAGAGAGCAC | 20 |
| miR157 | ath-miR157a-5p | UUGACAGAAGAUAGAGAGCAC | 21 |
|  | ath-miR157d | UGACAGAAGAUAGAGAGCAC | 20 |
| miR159 | ath-miR159a | UUUGGAUUGAAGGGAGCUCUA | 21 |
|  | ath-miR159b-3p | UUUGGAUUGAAGGGAGCUCUU | 21 |
|  | ath-miR159c | UUUGGAUUGAAGGGAGCUCCU | 21 |
| miR160 | ath-miR160a-5p | UGCCUGGCUCCCUGUAUGCCA | 21 |
|  | ath-miR160a-3p | GCGUAUGAGGAGCCAUGCAUA | 21 |
| miR162 | ath-miR162a-3p | UCGAUAAACCUCUGCAUCCAG | 21 |
| miR164 | ath-miR164a | UGGAGAAGCAGGGCACGUGCA | 21 |
|  | ath-miR164c-5p | UGGAGAAGCAGGGCACGUGCG | 21 |
| miR165 | ath-miR165a-3p | UCGGACCAGGCUUCAUCCCCC | 21 |
| miR166 | ath-miR166a-5p | GGACUGUUGUCUGGCUCGAGG | 21 |
|  | ath-miR166a-3p | UCGGACCAGGCUUCAUUCCCC | 21 |
|  | ath-miR166e-5p | GGAAUGUUGUCUGGCACGAGG | 21 |
| miR167 | ath-miR167a-5p | UGAAGCUGCCAGCAUGAUCUA | 21 |
|  | ath-miR167d | UGAAGCUGCCAGCAUGAUCUGG | 22 |
| miR169 | ath-miR168a-5p | UCGCUUGGUGCAGGUCGGGAA | 21 |
|  | ath-miR168a-3p | CCCGCCUUGCAUCAACUGAAU | 21 |
| miR169 | ath-miR169a-5p | CAGCCAAGGAUGACUUGCCGA | 21 |
|  | ath-miR169b-5p | CAGCCAAGGAUGACUUGCCGG | 21 |
|  | ath-miR169h | UAGCCAAGGAUGACUUGCCUG | 21 |
| miR170 | ath-miR170-3p | UGAUUGAGCCGUGUCAAUAUC | 21 |
|  | ath-miR170-5p | UAUUGGCCUGGUUCACUCAGA | 21 |
| miR171 | ath-miR171a-3p | UGAUUGAGCCGCGCCAAUAU | 20 |
|  | ath-miR171b-3p | UUGAGCCGUGCCAAUAUCACG | 21 |
| miR172 | ath-miR172a | AGAAUCUUGAUGAUGCUGCAU | 21 |
|  | ath-miR172c | AGAAUCUUGAUGAUGCUGCAG | 21 |
|  | ath-miR172e-3p | GGAAUCUUGAUGAUGCUGCAU | 21 |
| miR2111 | ath-miR2111a-5p | UAAUCUGCAUCCUGAGGUUUA | 21 |
| miR319 | ath-miR319a | UUGGACUGAAGGGAGCUCCCU | 21 |
|  | ath-miR319c | UUGGACUGAAGGGAGCUCCUU | 21 |
| miR390 | ath-miR390a-3p | CGCUAUCCAUCCUGAGUUUCA | 21 |
|  | ath-miR390a-5p | AAGCUCAGGAGGGAUAGCGCC | 21 |
| miR393 | ath-miR393a-5p | UCCAAAGGGAUCGCAUUGAUCC | 22 |
| miR394 | ath-miR394a | UUGGCAUUCUGUCCACCUCC | 20 |
| miR395 | ath-miR395a | CUGAAGUGUUUGGGGGAACUC | 21 |
|  | ath-miR395b | CUGAAGUGUUUGGGGGGACUC | 21 |
| miR396 | ath-miR396a-5p | UUCCACAGCUUUCUUGAACUG | 21 |
|  | ath-miR396a-3p | GUUCAAUAAAGCUGUGGGAAG | 21 |
|  | ath-miR396b-5p | UUCCACAGCUUUCUUGAACUU | 21 |
|  | ath-miR396b-3p | GCUCAAGAAAGCUGUGGGAAA | 21 |
| miR398 | ath-miR398a-3p | UGUGUUCUCAGGUCACCCCUU | 21 |
|  | ath-miR398b-3p | UGUGUUCUCAGGUCACCCCUG | 21 |
| miR399 | ath-miR399a | UGCCAAAGGAGAUUUGCCCUG | 21 |
|  | ath-miR399b | UGCCAAAGGAGAGUUGCCCUG | 21 |
|  | ath-miR399d | UGCCAAAGGAGAUUUGCCCCG | 21 |
| miR403 | ath-miR403-3p | UUAGAUUCACGCACAAACUCG | 21 |
| miR408 | ath-miR408-3p | AUGCACUGCCUCUUCCCUGGC | 21 |
| miR8175 | ath-miR8175 | GAUCCCCGGCAACGGCGCCA | 20 |
| miR858 | ath-miR858a | UUUCGUUGUCUGUUCGACCUU | 21 |
|  | ath-miR858b | UUCGUUGUCUGUUCGACCUUG | 21 |

**Table S2** **Identification of novel miRNAs**

| miRNA name | Sequence (5′-3′) | Length (nt) | miRNA name | Sequence (5′-3′) | Length (nt) |
| --- | --- | --- | --- | --- | --- |
| novel_1 | CUUGGACUGAAGGGAGCUCCCU | 22 | novel_10 | UUCCACAGCUUUCUUGAACUU | 21 |
| novel_103 | UGUACGGGUUCAUAGAUGA | 19 | novel_104 | UUUAAUUUCCUCCAAUAUCUCA | 22 |
| novel_105 | UGUGUUCUCAGAUCGCCCCUG | 21 | novel_106 | AUGGCUCAAAGCUGUUAAAACGAC | 24 |
| novel_107 | AUUUGUGUCGUUCGGAUAUCAAUU | 24 | novel_108 | UUUCCUAAACCACCCAUUCCUC | 22 |
| novel_110 | CGGGAAUCGUGAUGGGCCUCACUGG | 25 | novel_112 | AACAACGUCGUUUUGGUCAAUUUG | 24 |
| novel_113 | AGAACUUUUGGUAGAAUUUUUGGG | 24 | novel_114 | ACCGGUCAUGUCUUUGUUAUGAUU | 24 |
| novel_116 | AUUUUGAUGAUCUAUUCUGUCCAU | 24 | novel_119 | UUUCCUAUUCCUCCCAUACCUA | 22 |
| novel_12 | UUGCAUACGCACCUGAAUCGG | 21 | novel_120 | AUCUGAUUCGAUUUGGUUUCAAACC | 25 |
| novel_121 | GGAAUCUUGAUGAUGCUGCAG | 21 | novel_123 | AGUUUGUAUUUUGCGUCACUUUGG | 24 |
| novel_126 | CACGUCGGUGAUUUUGGUGAU | 21 | novel_127 | AAGUGACUGUAACAACAACGUUGC | 24 |
| novel_13 | UCGGACCAGGCUUCAUUCCCC | 21 | novel_132 | UCUCGGACCAGGCUUCAUUCC | 21 |
| novel_133 | AAGGGUUACAAAACUUUCAUGAUU | 24 | novel_135 | UUCCUCAACUAUGAAUUCGUACAU | 24 |
| novel_138 | AAAAUGAGUUGGACCCCCUCAAGG | 24 | novel_139 | AUAAAUUUCCGAGAACUCUUUAGG | 24 |
| novel_14 | UUCCACAGCUUUCUUGAACUG | 21 | novel_141 | AGUGUCCGAUAUUAUGAAUUUA | 22 |
| novel_143 | ACACGUGGCGGGUGGUGAUUGGAG | 24 | novel_144 | UUGUGUUUUAUUUCUGUACAAGUA | 24 |
| novel_146 | AGGAUUACUUUUCUGUAUCUGAGU | 24 | novel_147 | AUUCGAGAUUUGAUAUGGACGUGU | 24 |
| novel_16 | UUUCCAAGUCCACCCAUUCCUA | 22 | novel_18 | UGGCAAGCUGCUGAUCCUAUC | 21 |
| novel_20 | AAUCUCCCGAACUAUAUUCUUU | 22 | novel_21 | UAAAUGCGAUCCCUUGGGAAU | 21 |
| novel_23 | UGUCGCGGAUAAUUUUGGCACA | 22 | novel_24 | UUCCCUACGCCACCCAUUCCGU | 22 |
| novel_27 | GAAAGACUUUUCAUGAUACCUAGC | 24 | novel_28 | AUGCUCUUCUGAACUGUGCAGUGA | 24 |
| novel_30 | UAUUGGCCUGGUUCACUCAGA | 21 | novel_31 | UUUAGUUUCCUCCAAUAUCUUA | 22 |
| novel_32 | UACGGGUUCAUAGAUGAAGAAU | 22 | novel_33 | UCUUGCCCAGACCUCCCAUACC | 22 |
| novel_34 | UGUAAUUAGUAGUUCUACCACUU | 23 | novel_35 | AAUGUGAUUGGUUUAUAUAGUGGG | 24 |
| novel_36 | CUGAAGUGUUUGGGGGAACUC | 21 | novel_37 | UUUUGAUAAUCUGAACUGUUC | 21 |
| novel_38 | AUGGUAGAGAAGUGGUUUGGGACA | 24 | novel_39 | AAUGUGUAGAAAUCGUCGAGUAUU | 24 |
| novel_44 | ACGGGGACGAGACAGAGCAUG | 21 | novel_45 | CAUGUGCCCCUCUUCCCCAUC | 21 |
| novel_46 | AUUCAAGAAUGUGUGUAGAGA | 21 | novel_48 | ACAAGCCGGUUGUCCAGGAGGAUG | 24 |
| novel_49 | UUCCCUAAUUGAUGUCGGACAAUU | 24 | novel_50 | AUUGUAAGGUUCGUCGAAUAGAUC | 24 |
| novel_52 | GCUUCCGUGCUAAGAUAUUCUUUAC | 25 | novel_54 | CGUGCUGUCUAUCGUCGUCAU | 21 |
| novel_55 | CGAUUAUGUAAAGCCAAAAGC | 21 | novel_56 | CACGUGCUCCCCUUCUCCAAC | 21 |
| novel_57 | GAAAUUAUUAGGUAGUCUCGGACA | 24 | novel_58 | AAGUUAGUUUGUUUGGCAUAUUCU | 24 |
| novel_6 | UCGGACCAGGCUUCAUUCCUC | 21 | novel_62 | UACGGUCUAUCCCACGCUAAU | 21 |
| novel_68 | AGACUAUAUGGUUCGGAAAACAACU | 25 | novel_7 | UUUGGAUUGAAGGGAGCUCUA | 21 |
| novel_70 | UACGGGUUCAUAGAUGAGGAG | 21 | novel_72 | ACAUUCGACGUGGCCAUGUACUGG | 24 |
| novel_73 | ACAGAAAUCAAUCACAGAAUUAGA | 24 | novel_74 | CAUUAAUUACUGUAUCAAUUA | 21 |
| novel_76 | AAGUCGACACGGUAGAGCCAAGAC | 24 | novel_78 | AUGACAGAAGAGAGUGAGCAC | 21 |
| novel_79 | ACCGGCAUUAGCUUCGACACAUG | 23 | novel_8 | UGAAGCUGCCAGCAUGAUCUGA | 22 |
| novel_81 | AGUGUCUGAUAUUAUGAAUUUA | 22 | novel_82 | GGGAUUGUAGUUCAAUUGG | 19 |
| novel_83 | UGGAUGUAGCAAAGAGAAGCU | 21 | novel_84 | AUUUCUUGGUUGCUUUACUCC | 21 |
| novel_85 | AAUGCUCUGAUACCAUGUUAA | 21 | novel_86 | AAAUGGCUCGAAUGUCUCAAAACG | 24 |
| novel_88 | GGUUAACAUUGUUGCUAACUGUGG | 24 | novel_89 | UGGGAAUCCUGAUGAUGCUGC | 21 |
| novel_90 | AUGGCUCAAAACUGUCAAAACGAC | 24 | novel_91 | GAGCACCAUAUGACCUAUGCCACG | 24 |
| novel_92 | UGAAGCUGCCAGCAUGAUCUAG | 22 | novel_93 | UUGUGUUUGGAUCAUAGAUUU | 21 |
| novel_94 | AAAACAUGUUGUUUGACUCCUAGA | 24 | novel_96 | UUUUGUUCGGAAGUUGAUAUC | 21 |
| novel_98 | ACGAUCUAAACUGUUUAUCUUGUG | 24 | novel_99 | AUGGCUCAAAACUGUUAAAACGAC | 24 |
